# A needs-based approach to promoting gender equity and inclusivity: insights from participatory research with farmer-producer organisations (FPOs)

**DOI:** 10.1007/s40847-023-00280-x

**Published:** 2023-11-23

**Authors:** Tomás Harrington, Nivedita Narain, Nitya Rao, R. Rengalakshmi, Reetu Sogani, Shuvajit Chakraborty, Astha Upadhyay

**Affiliations:** 1 University of East Anglia, Norwich, UK; 2 PRADAN (Professional Assistance for Development Action), New Delhi, India; 3 M S Swaminathan Research Foundation, Chennai, India; 4 Lok Chetna Manch, Ranikhet, Almora, Uttarakhand India

**Keywords:** Farmer-producer organisation (FPO), Women farmers, Individual needs, Collective spaces, Gender equity, Inclusive development, India, Q01, P13, J24

## Abstract

The farmer-producer organisation (FPO) is an umbrella term used to describe modes of farmer collectivisation in India, i.e. co-operatives and companies. While women cultivators play a central role in agriculture, their continued marginalisation is reflected in a lack of engagement in FPO activities and governance structures, with only 3% of existing FPOs being female-led ventures. This paper examines the nature of tensions around social inequities—and how such tensions might be addressed in the collective spaces offered by FPOs—using a gender lens, specifically in balancing conflicting pressures of economic performance versus inclusion and meeting specific members' needs. Using a participatory research approach, a conceptual framework is developed and applied in three FPOs operating at various stages of development. With a specific focus on gender equity and social inclusion, selected cases involved FPOs with > 75% female membership and scheduled caste/tribe participation. Qualitative data on societal needs, based on the expectations and experiences of FPO participants, are then analysed to better understand (1) what might promote FPO participation and (2) how equity and inclusion may be enabled from the ‘bottom-up’. This exploratory study informs the collective action debate with its new and intersectional approach to gender equity and inclusivity. Empirical observations and within-case analyses involving FPOs provide new insights into the functioning of these institutions and nuanced interactions involving their members. Findings suggest that informal micro-producer arrangements or *vyavastha*, in the FPO pre-registration phase, are well positioned to act as spatial agents for establishing ethical norms as FPOs collectivise and grow. In terms of promoting social objectives and evaluating FPOs operating in different stages, a set of guiding principles are proposed with follow-on implications for policy.

## Introduction

While India has had a long history of successful co-operatives in sectors such as dairy, sugar, handloom and poultry (Kumar 2010; Shah 2016; Ashok 2017; Prasad and Gautam 2019; Agarwal 2020), many experiences—especially in joint cultivation—have been less than impressive (Agarwal 2010). Failures have often been attributed to ‘top-down’ approaches (Jain and Coelho 1996), where initiatives have neglected the basic needs of individual members. With an emphasis on economic return, little attention is paid to critical social aspects, involving marginalised and less powerful members in these collectives, compromising their credibility in the process (Shylendra 2021).

Persistent issues, linked to the security of land tenure, access to markets and a stagnation in rural incomes, have led to a recent emphasis on the ‘farmer-producer organisation’ (FPO) as the solution to this rural impasse (Govil et al. 2020). The FPO is an umbrella term used to describe modes of farmer collectivisation in India, encompassing both co-operatives and companies (FPCs) (Kumar et al. 2022). It is estimated that 7000 FPOs—with 4.3 million small producers contributing towards their share capital—are now operating across India (Neti et al. 2019). While there has been a rapid increase in numbers since 2014, due to support from government schemes and subsidies, more than 50% of these are concentrated in just four Indian states and specific districts close to megacities and industrial hubs. A majority of these FPOs are also in the very early stages of their operations, with paid-up capital of less than INR 1 million,^1^ limiting possibilities for growth, generating returns for shareholders or becoming viable only in the longer-term (Neti et al. 2019; Kumar et al. 2022). While assessment here appears purely economic, the state has been promoting FPOs with funding to achieve ‘social objectives’. This raises ethical questions not just about the criteria for FPO assessment, but equally a responsibility to ensure that both state funds, and the meagre contributions of small producers, are used effectively to generate returns—economic, social and political.

This paper examines the nature of tensions around social inequities and how such tensions might be addressed in the ‘collective spaces’ offered by FPOs. Specifically, it examines the balancing of conflicting pressures of economic performance versus social inclusion and meeting the needs of the less powerful in a collective. Using a gender lens—this study focuses on the experiences and expectations of women who play a central role in cultivation in developing countries (Fuchs et al. 2011), but whose continued marginalisation is reflected in a lack of involvement and engagement in FPO formation activities and governance structures. With the Government of India publishing detailed guidelines for the creation of 10,000 new FPOs in India by 2024 (Sinha 2020), women-led FPOs are projected to make up only 5.9% of the 17,000 FPOs envisioned (Vasavada 2021)—a significant arena of inequity.

### Research gaps informing research questions

While many needs-based approaches assess human rights and perceptions of life quality (Janker and Mann 2020), a research gap exists in studies of producer-based organisations. Despite the emergence of collective action (CA) research involving FPOs (Trebbin and Hassler 2012), there is little empirical evidence on how CA models can help women farmers overcome gender-specific challenges in terms of engagement and sustainable livelihoods (Baruah et al. 2022). Empirical research that examines inclusivity and the role of women in agriculture, specifically, their involvement in FPO formation and governance are also scarce in India (Dohmwirth and Hanisch 2019; Vasavada, 2021), given that only 3% of FPOs are female-led ventures (Neti and Govil 2022).

While many FPOs look to counter exclusion, a ‘*common theoretical perspective on inclusiveness is still lacking*’ (Bitzer and Marazzi 2021: 381). In this study, we seek to understand what might promote participation in FPOs, in the context of women farmers who are often side-lined. We specifically focus on ‘new forms of participation’, transitions where women have been empowered to engage in FPO value addition processes, unrestrained by socio-cultural norms.

Of the 7000 FPOs in operation in 2019, the majority remain ‘stuck’ at early stages of operation, unable to meet basic (financial) compliance requirements. In practice, FPOs also remain beset with issues in meeting specific members' needs, as social objectives and measures are lacking and may be unique at different stages of FPO development and operation.

While drawing on Indian case studies, this paper has wider global relevance in this regard. Motivated by these gaps, we formulate the following two research questions:

- **RQ#1**—How might ‘new forms of participation’, capturing the interplay between individual needs and collective spaces, be best represented?
- **RQ#2**—What principles might best guide FPO design and assessment at various stages of development, from a gender equity and inclusion perspective?

In addressing RQ#1, we develop a conceptual framework to elaborate on the idea of FPOs as a collective-shaping continuum, where inclusivity and members’ needs may be dynamic and dependent across different stages of FPO development. In addressing RQ#2, the conceptual framework is used to unpack a series of guiding principles based on a participatory research approach involving three female-led FPOs.

The paper is structured as follows: The next section outlines the conceptual framework development. The methodologies employed are outlined in Sect. Methodology. We summarise three case studies, empirical observations and within-case analyses, in Sects. Case studies and Empirical observations, respectively. Section Discussion outlines a discussion based on the conceptual framework. The paper concludes with policy implications, study limitations, and future research plans.

## Conceptual framework development

To explore the spatial and social dynamics of FPOs, we focus on both processes that might allow *freedom* of actions and decisions, and actual *opportunities* that participants expected and experienced (given individuals’ social contexts) (Sen 1999).

### Conceptual framework: components and interactions

We position individual needs and collective spaces as being central to equity and inclusion. Alderfer's dynamic conceptualisation of human needs (1969) served as the basis for elaborating and unpacking different ideas and approaches to FPOs, in explaining how the satisfaction or frustration of a need might motivate actions, which may, in turn, lead to meeting that need or other needs. This aspect of the theory, namely the actions taken, appears especially relevant for the stages of emergence explored in this study. This is so because (1) it describes societal needs met/not met by these new institutional arrangements but also (2) the transition to this point (de Haan et al. 2014). The theory assumes three core categories of individual needs: existence; relatedness and growth (ERG). In summary:

- Existence needs are concerned with health and well-being and basic needs such as material and physiological desires, working conditions and pay;
- Relatedness needs are those that involve relationships with other individuals and one’s environment. Relatedness has also been described as a facilitator of social interactions and, hence, interpersonal relationships (Johnstone et al. 2012), reflecting the extent to which an individual feels connected and belonging to a community, e.g. social cohesion
- Growth needs relate to the creative and productive effects of both the individual and collective agency/action on the individual and their environment, e.g. purpose and expression, influence and respect, freedom and autonomy.

These needs, however, are gendered, reflecting both everyday material needs, and more strategic ones, linked to social status and positioning (Molyneux 1985; Rao 2017a, b). Gender relations  are contextual, reflecting differences in labour divisions, asset ownership and social norms, across time and place, similar is the construction of needs. Collectives provide women with space to establish the legitimacy of their needs and seek satisfaction (Fraser 1989). In doing so, they facilitate a process of questioning unequal power relations and can set in motion a process of empowerment.

In terms of stages of emergence, the extant literature offers organisational life cycle stages and phases from multiple perspectives (Harrington and Srai 2017): those that include start-ups; adolescence and growth; and maturity and decline (and most overlap to some extent). There is also a consensus in conceptualising these stages or transitions, as clusters of predictable tensions (Quinn and Cameron 1983; Narain et al. 2020). Identifying these stages of FPO development allows the leadership (and those supporting the growth and development of FPOs), to anticipate, recognise and respond to the predictable clusters of unmet needs (or tensions) and better enable transitions across stages. Hence, we make a link here to objective criteria for case selection (see also next section):

- The nascent stage refers to FPO activities that may be uncoordinated in terms of organisational structure, product definition, formal systems and processes (i.e. a registered FPO but not functional; an initiated process of group formation, where informal arrangements exist but not yet as a formal legal entity). This stage aims to capture critical activities that take place pre-registration (e.g. cluster analysis and the organisation of farmer interest groups) as outlined by the Indian Ministry of Agriculture Policy and Process Guidelines for FPOs (Raju et al. 2017).
- The emerging stage captures those critical activities that take place in the post-registration phase. It broadly covers the transition from viable pilots to a completed value chain with end-users and early adopters, with a focus on defining market requirements and delivery channels for a new offering.
- The mature stage refers to the development of a viable business concept where a self-reliant FPO may have the potential for scale-up. Organisations at this stage are often in a state of flux as supply network elements (activities; actors) undergo rapid and continual change as associated value chains evolve (Harrington and Srai 2017).

**Fig. 1:**
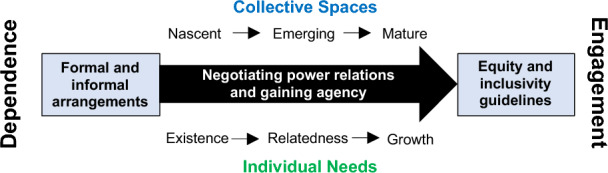
Conceptual framework linking individual needs and collective spaces

We define ‘collective spaces’ as networks of support provided by self-help groups (SHGs), village organisations (VOs) and more formal FPOs or federations of groups. Here, it is analytically useful to distinguish between what Cox (1998: 2) calls the ‘spaces of dependence’ which reflect localised social relations key to meeting basic existence needs, and ‘spaces of engagement’ involving the external environment outside of the immediate ‘spaces of dependence’. Our conceptual framework (Fig. 1) aims to specifically capture how these ‘spaces’ might affect smallholder farmers, especially women, as it is their social identities (ethnicity, resource access and gender) that mediate relationships within and across these spaces, with wider networks of stakeholders.

In summary, the conceptual framework illustrates interactions between ERG theory (individual needs) and FPO stages of emergence (collective spaces) to address RQ1. These components are used to operationalise participants’ reflections at various ‘stages’ of FPO development in research phase 2 of this study.

## Methodology

**Fig. 2:**
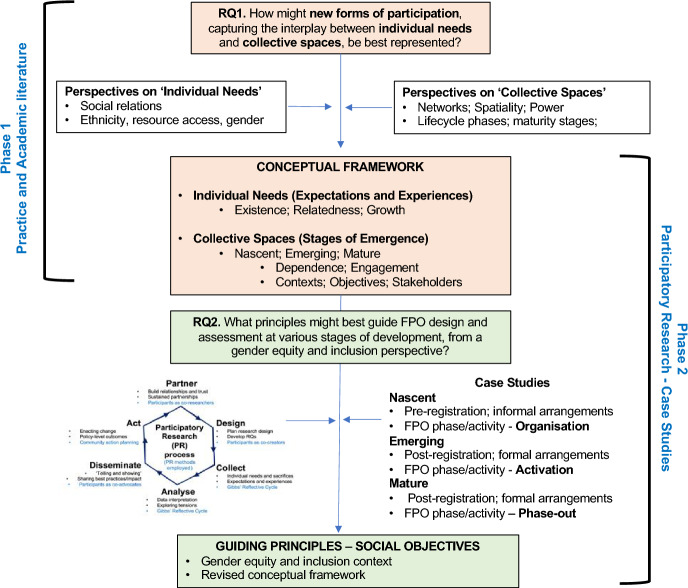
Methodology and phases of research

A literature synthesis was used to inform the development of the conceptual framework and address RQ1. A case study methodology (Yin 2009) was then used to address RQ#2. Figure 2 summarises the two phases of research in this study.

In phase 2, a participatory research (PR) approach was adopted to support a within-case analysis (see Appendix 1 for a summary graphic of the PR process and interview protocol). PR approaches differ from more traditional social science methods in that they are based on reflection, data collection, and action (Vaughn and Jacquez 2020). It is also important to note that a crucial step in PR involves sustained partnerships, where relationships and trust have been built between researchers and participants over several years. PR is particularly suitable for this study: it involves some of the most marginalised communities in India and enables their ‘voice’ to be heard. It thus offers much deeper insights into the functioning of institutions and nuanced interactions involving its members.

**Fig. 3:**
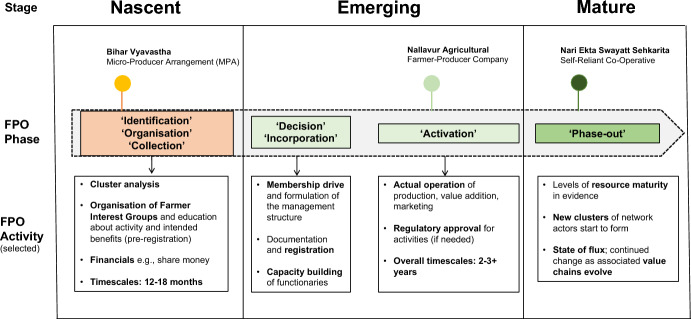
Positioning of cases based on FPO phase activities and observations. Stages and phases adapted from Raju et al. (2017) and Harrington and Srai (2017)

Three cases were selected from existing field studies. Criteria for case selection followed a clear, structured and consistent process:

1. *FPOs at different stages of development*: 12 cases were initially identified based on objective criteria set out in the previous section and Table 1.
2. *FPOs that were female-led*: Based on specific FPO processes (see Fig. 3), three FPOs, with > 75% women participants were identified:
  1. A nascent case in the advanced stages of FPO initiation (pre-registration);
  2. An emerging case, post-registration, which had successfully progressed through the nascent FPO stage;
  3. A mature case in ‘phase-out’ which was self-reliant, having progressed successfully through nascent and emerging FPO stages.
3. *FPO member profiles*: Among FPO members, 37 participants were randomly selected (male/female; initiators, trainers, adopters) who were promoting inclusion activities. Some 60% of participants were involved in greater than two FPO stages, which helped with data triangulation efficiency (Yin 2009).

**Table 1:** Case contexts, selected demographic characteristics and overview of participants

Case	Bihar Vyavastha Micro-producer arrangement (MPA)	Nallavur Agricultural Farmer–Producer Company	Nari Ekta Swayatt Sehkarita Nari Ekta Self-Reliant Co-Operative
Stage of emergence	**NASCENT**	**EMERGING**	**MATURE**
Registration status	Pre-registration	Registered as per Amended Companies Act (2012)	Registered as per the Co-op Societies Act
Years of Operation (informal and formal)	Informal associations/arrangements in place since 2018	7 years; Farmer-Producer Company formally registered in 2015	15 years; formed in 2007
FPO phase and activities (adapted from Raju et al. 2017)	**‘ORGANISATION’** Post-‘Identification’ and Pre-‘Collection’ activities at the Nascent stage	**‘ACTIVATION’** Post-‘Decision’ and ‘Incorporation’ activities at the Emerging stage	**‘PHASE OUT’** Self-reliant in terms of operations and many activities
Value Chain Development (adapted from Harrington and Srai 2017)	**Embryonic/Fragmented**—Little value network structure; Weak product definition; Issues around business planning and primary processing	**Formation**—Transitioning from viable pilot schemes (trials) to complete value chains with end-users; Issues around forward and backward integration; Critical activities include trust building and selection processes	**Stabilisation**—Clusters of network actors starting to form their own initiatives; Issues around fragmentation of the original network
State (block; district)	**Bihar** (Chakai block; Jamui district)	**Tamil Nadu** (Mailam block; Villupuram district)	**Uttarakhand** (Bhaisiyachana block; Almora district)
Cluster/ Panchayat	**3 Panchayats** (81 villages)	**12 Panchayats** (20 hamlets)	**19 Gram Panchayats** (33 revenue villages; > 100 hamlets)
Size (membership)	** ~ 1000 members** (70% women; 30% Young Males (16–40);	** ~ 1000 members** (84% women; 16% men)	**1336 members** (87 SHGs where members are 100% women; Shareholder ratio: 82% women;18% men)
Number and details of participants (summary)	**15 participants** 53.3% female; 46.7% male 33.3% aged > 40	**12 participants** 83.3% female; 16.7% male 41.7% aged > 40	**10 participants** 80% female; 20% male 60% aged > 40
Roles and membership—participants	46.6% initiators and trainers/mentors; 53.3% adopters	58.3% leaders/directors; 41.7% members	100% shareholders

The conceptual framework was used to develop a semi-structured interview protocol, to gather primary data. The interview process consisted of two steps: step 1 involved a pilot to test and refine the structure and wording of the interview questions; step 2 involved data collection through in-depth interviews which took place from March to December 2020. Ethical considerations and interview questions are outlined in Appendix 1.

The conceptual framework, in linking collective spaces and individual needs, was also used to organise the within-case analyses. Given the exploratory nature of the study, this within-case approach was designed to report rich and detailed data that best captured real-world contextualisation. Reflections from participants were translated and transcribed by the authors. The interview transcripts, and individual case summaries, were collectively reviewed and then systematically grouped into themes and sub-themes (existence; relatedness; growth). Data were also clustered in terms of stages (nascent; emerging; mature) to gain better clarity on conceptually similar interactions. A qualitative content analysis was then used to process information relevant to the refinement of the conceptual framework (Kohlbacher 2005).

## Case studies

Sections Bihar Vyavastha Micro-Producer Arrangement (MPA) – nascent case–The Nari Ekta self-reliant co-operative: mature case provide a brief overview of the three cases. Figure 3 provides a visual representation of the cases, based on stages and activities, which served as objective criteria for selection. Table 1 summarises case contexts with selected demographic characteristics.

### Bihar Vyavastha micro-producer arrangement (MPA): nascent case

The Bihar Vyavastha Micro-Producer Arrangement (MPA) is at a nascent stage of operation where critical activities in 2020 involved the organisation of Farmer Interest Groups (i.e. the ‘organisation’ phase pre-registration). MPAs can be best described as informal associations that support farmers to federate into a producer organisation at a later stage. The overall vision is to build a local ecosystem of service providers and market actors and an inclusive farmers' and local traders' organisation, to strengthen the village economy (statement from NGO PRADAN, the facilitating agency). Based in South Bihar, the poorest of the three sites, MPAs have tended to be organised within the site’s SHG network to leverage the strengths of the network to enhance incomes and engage previously excluded landless workers. Participants include ‘initiators’, who instigated the *vyavastha* or MPA in their villages and neighbouring areas, engaged women SHG members and VO office bearers (e.g. in cauliflower cultivation as a high-value commercial farming venture; providing much-needed market linkages by ferrying produce to markets and charging a small commission). Participants also include ‘adopters’ who benefitted from MPAs providing the working capital to cultivate new crops, de-risking their sale in unknown markets.

### The Nallavur agricultural farmer-producer company (FPC): emerging case

The Nallavur Agricultural Farmer-Producer Company is at an emerging stage of operation with actual production and marketing in the ‘activation’ phase. Situated in the Mailam block of the Villupuram district in Tamil Nadu, about 90% of farmers are marginal landholders owning < 1 ha of land. The FPC aims to focus on equal opportunities and women’s participation and access to productive resources. Women have been mobilised and organised into SHGs here since the early 2000s: 400 groups are active out of the 526 formed. A cross-section of group members from different cadres of the Nallavur FPC were randomly selected to understand their different perspectives and experiences. Eleven participants have been part of the FPC for > 5 years, mobilised as part of the Nallavur project partnership with the NGO MSSRF, promoting the production of pulses for enhanced livelihoods. Single women households, and smallholders from socially disadvantaged backgrounds, were included as initial members.

### The Nari Ekta self-reliant co-operative: mature case

The mature case study is situated in the Bhaisiyachana block of Almora district in Uttarakhand located in the mid Himalayas. More than 90% of the farmers here too are small and marginal holders, practising mostly rainfed agriculture. The current aims of Nari Ekta Swayatt Sehkarita include: improving access for marginalised women to technical and business services, conducting activities for social and economic upliftment of community-based groups and individuals, and promoting small-scale business development. Participants were representative of the 82%:18% shareholder ratio of women to men; in terms of SHG members, 100% are women. All participants practice a type of mixed farming: growing millets, wheat, paddy, vegetables, spices, fruits, oil seeds, and lentils. The focus of the FPO is predominantly to support subsistence women farmers, with small surpluses available for sale. Members have access to seeds and farm machinery, and information on new practices including organic farming (using biopesticides), made available through the research institution, VPKAS (*Vivekanand Parvatiya Krishi Anusandhan Sansthan, Almora*).

## Empirical observations

Selected within-case observations are now presented and then summarised. In each stage, participants reflected on their expectations and experiences involving the three categories of needs—existence, relatedness and growth.

### Nascent case observations

The majority of respondents prioritised earning an income for self and family as existence-related needs, which was not possible without bringing others on board (e.g. with knowledge about quality requirements and meeting market needs). But even during this phase, emerging relatedness and growth needs, around working capital, absence of forward and backward linkages, inadequate business planning and primary processing were acknowledged, especially by the initiators or leaders. They saw such elements as instrumental in supporting existence-related needs rather than being of intrinsic value. Yet, with membership still fluid, and decision-making mechanisms not clear, the arrangements were still evolving, with farmers thinking through next steps as they tried new activities and ventures.

Women respondents reflected on how they initiated the *vyavastha* with the help of male farmers, PRADAN, and agricultural trainers, to strengthen their livelihoods. While both women and men initiators were motivated by their family existence needs, they were quick to realise that the success of a larger grouping and their own family goals were interlinked.

In terms of relatedness needs, a sense of identity, self-confidence and equality involving the Santal group members was an important change, in not just their ability to farm, but also to link to markets and gain remunerative prices: “*A few years ago…non-Santals grew vegetables but we did not…it helped us in realising what we could do to use our land…now we can sell too*”. As more farmers joined the *vyavastha* (arrangement)*,* they hoped that their voice would be heard at an institutional level: “*One farmer will not be able to access…but when we are recognised as a collective, government schemes would reach us…”.*

The MPA appealed to women for other reasons—it saved time and helped overcome mobility restrictions. Despite some initial doubts, ‘Chhoti’ spoke in positive terms about transformation: “*…before we started working in a group, nobody gave any importance to my views, but after joining the group people started listening to me…”.* The experience of selling produce together in the market enabled them to make more profits than they had ever earned before. It unexpectedly met the need to learn from each other, particularly new techniques of farming: *“…nobody knew about medicines and vaccinations…now I am trying to persuade other women also; so they know that collectively we can form a stable group where all these facilities will be available for all the members”* (‘**Jyoti’**).

### Emerging case observations

In the emerging case, there was greater articulation of relatedness and growth needs across both leaders and members. Participants spoke about the need for continuous engagements (in terms of exposure visits and other activities) with members to ensure the active status of the group at a grassroots level. ‘Geetha’ also commented that the demonstration of successful trials in the region ‘*helped me gain confidence to join the group despite initial resistance from my family members’*.

In subsequent years, priorities and needs have expanded, which respondents mainly attributed to their exposure and interaction with other members and outside experts who came for specialised training. The group approach helped them to expand/strengthen their networks and discussion spaces focusing on livelihoods and social issues. As a result, members’ ambitions changed and needs began to incorporate more social as well as economic dimensions, reflected in respondents now aspiring to become group leaders and board members. ‘Prabhu’ commented, after conducting the annual general body meeting in which more than 800 members participated: “*Now as an active board member, through this identity, I receive respect among relatives, networks, and local authorities*”.

Due to a lack of land titles, women had earlier been denied access to agricultural credit and other benefits. Women’s space has often been confined to their homes and villages—the ‘domestic domain’—working in the fields to feed their families yet denied entitlements and access to services elsewhere (Rao 2012). In terms of growth needs, group discussions were conducted during the initial phase of the Nallavur project which helped identify gaps in current production practices and access credit support from banks and government entitlement schemes. The evolution of their organisation has changed outlooks and attitudes—the concept of ‘women farmers’ slowly emerged and women’s ability to manage and lead the farm operations is accepted by their households and external stakeholders. As a result, women members expressed their confidence in farming independently and also expanding their cultivation by leasing land: *“…I received additional services from the group…Access to such services might not have happened if I was an individual farmer. Because I am in the group with other women members, I can go and attend the training and interact with external persons and institutions, otherwise as a woman, I can't do that, my family members may not encourage such actions"* (**‘Geetha’**).

### Mature case observations

In terms of existence needs, respondents had become aware of what women and men had achieved through increasing exposure and interactions with a wider network. Group savings were especially beneficial as they had enabled women to become self-sufficient in not having to rely on banks for loans when their savings reached Rs.150,000. Additionally, group savings had also given women a sense of security, inculcated a sense of self-respect, and helped many families pay off their loans. Women also noted the need to support those weaker than themselves, especially single women.

Beyond the group, they saw the federation as an important space for engaging with external institutions, gaining new information, knowledge and indeed resources. Access to scientific research institutions like VPKAS, training and interactions with external agents, and the contributions this had made to business promotion, was a very important part of the experience of relatedness. Social recognition has meant that people from outside now come to tell them about government schemes and bank loans.: “*Joining the federation helped us learn new techniques like poultry farming, compost making, and biopesticide preparation****.**** The training has enabled us to regularly prepare and use these products. We have met so many new people from different places. It taught us so we are now capable of earning our bread and butter*” **(‘Guna’)**.

In terms of growth needs, the collective has enhanced a sense of self for women, giving them a social identity beyond their homes. It has also given them immense self-confidence—a sense of power within, but also a power to act: “*It is not easy. I fought with my husband for many years as he would not let me come out of the house. He still stops me but I don't listen to him anymore. I really wanted to move ahead in life…*” (‘**Khili’**). This new confidence has also enabled several women to participate actively in local political processes. ‘Hiruli’, recently elected as *Gram Pradhan* (leader of the elected local government), acknowledged the support of the women's group and federation: "*it wouldn't have been possible otherwise"*.

### Summary of within-case analyses

In the nascent case, respondents spoke of how group formation had begun to enhance and sustain livelihoods, where farmers got more respect compared to when they were individuals. All the women and men interviewed were keen to establish an identity for their village in the markets and beyond, as part of their growth trajectory, so that people from the outside would recognise them as ‘*good farmers*’ and excellent producers of specific crops. While connecting with other farmers, to the government or market actors was explicitly identified by the initiators, the expectations were more subdued among some of the adopters due to issues of unequal participation (“*not everyone puts in the same labour*”). This poses an ongoing challenge to initiators, as they continue to mobilise and increase membership.

For the emerging case, five existence-related expectations emerged from interviews and within-case analysis: access to knowledge and training services to improve agriculture production; generating higher incomes; accessing credit and government schemes; expanding their social network; and gaining social identity. A central role of the FPO at this stage appears to be as an intermediary to various government departments, to access inputs, extension services, technologies and also credit. As well as positively impacting incomes, the FPC enabled respondents to expand their networks and contacts beyond village settings, by interacting with a range of government functionaries to secure their entitlements. Here, collective interactions and mutual trust over time are playing a central role in current value chain development activities (i.e. trust building and selection processes with suppliers). There appear to be specific challenges at this stage: First, incomes to meet the administration/management costs, since the profit margin generated was not sufficient to appoint quality HR resources; and second, given the inadequate working capital to facilitate the business, members’ contributions to the arrangement became increasingly important (cf. Neti et al. 2019). This issue of capital contribution by members, however, raises issues around ownership and control of the institution, as well as the perceived benefits.

The mature FPO case had greatly benefited from several years of a secure market contract through a governmental programme for the supply of nutritional foods to young children, as well as for pregnant and lactating mothers. While groups experienced some difficulties, in the timely planning and collection of produce or an inability to reach out to all members to purchase their surplus produce (due to delayed reimbursements from the state departments, affecting their working capital), they now recognised the importance of maintaining momentum and compensating for this through continuous engagements. Yet this was becoming increasingly difficult to maintain, and several inactive groups had already moved to constitute SHGs under alternate state schemes at the block level. A decline in numbers had implications for the strength of the FPO, as member contributions provided the capital to hire people to administer the FPO and build its business networks. Future policy moves had also required the arrangement to start thinking about developing governance and business models that might enable the FPO to compete with traders and agents, outside of their secure market with the state government.

Collectively, the analyses and supporting commentaries provide nuanced insights into the functioning of FPOs as institutions and interactions involving its members. Table 2 summarises the within-case analyses. Given space constraints, Appendix 2 provides commentaries on discriminating features across the cases.

**Table 2:** Summary of *within-case* analysis in terms of existence, relatedness and growth needs at different stages of emergence

	Nascent Bihar Vyavastha Micro-Producer Arrangement (MPA)	Emerging case Nallavur Agricultural Farmer-Producer Company (FPC)	Mature case The Nari Ekta Self-Reliant Co-Operative
Existence	Initiators focus on building collective solidarity at the local level and using this to leverage external spaces of engagement, in markets or through promoters (e.g. PRADAN); Adopter farmers focus on better prices and earning better incomes, and responding to initiator efforts.	Initial expectations centred on mechanisms to improve farming practices and enhance farm incomes and livelihoods; Needs have evolved—attributed to exposure and interaction with other members and outside experts (e.g. specialised training); New aspirations—growing confidence in members wanting to become group leaders.	Positive and visible change triggering a sense of security and confidence; Recognition of the importance of continuous engagements (e.g. in terms of training) to ensure the active status of the group at a grassroots level.
Relatedness	Relatedness focus is at a group level and vis-à-vis engaging with markets (bargaining collectively therein); Next steps—in establishing links with the state and securing benefits to expand enterprises identified Most initiators articulated relatedness needs despite scepticism amongst adopters.	Focus on expanding networks and spaces of engagement beyond the village through interactions with a range of government functionaries to secure entitlements.	Compensating against inactivity, irregular interactions, and decline in numbers (implications for FPO strength); Interactions with external agents a critical focus of relatedness, in this stage, in building business and societal contacts.
Growth	Whereas all the initiators articulated growth-related needs, for adopters it was more about being recognised.	The evolution of the network has changed outlooks and attitudes—the concept of ‘women farmers’ has slowly emerged and is now strongly integrated into group-related discussions.	Growth needs fuelled by insecurities (e.g. policy discussions may replace the distribution of nutritional foods with direct cash transfers to identified households); Future survival is likely to depend on market competitiveness and flexibility to adapt final product base.

## Discussion

We now unpack a series of guiding principles, based on the real-world application of the conceptual framework. Given their different stages of development from nascent in Bihar to mature in Uttarakhand, with Tamil Nadu in between, and recognising contextual differences across these sites, the conceptual framework helped capture some commonalities in terms of individual needs (i.e. shared expectations and experiences across a diversity of FPO stages).

A distinct transition was observed in terms of existence needs: from concerns around food security, cash for exigencies and employment to enhancing savings, capital formation and stabilising incomes in the nascent case, to incomes and improved practices in the emerging, and issues of autonomy and self-reliance in the mature arrangement. Some of these dimensions were also reflected in relatedness and growth needs and the spaces of engagement they sought out. Here, one observed that after more than a decade of participation in federation-type engagements, growth needs were not so much expressed in terms of income or employment, but more in terms of social and political recognition as equal and capable citizens. This recognition is a very important contribution of the FPO, yet it is hardly recognised in assessments of their profitability or indeed viability.

Relatedness and growth needs were also expressed, with an external focus, in the emerging FPO case. While they now recognised their skills in production, they expected support for marketing their products, and the need to compete with traders and wholesale market agents in terms of their business transactions. They had a future goal to establish their own brand name for certified seeds of black gram and groundnut and be a leader in delivering quality seeds of locally suitable varieties to farmers. Apart from dealing with the market, making themselves competitive implied reducing costs of production through appropriate training and support to run custom hiring centres that could provide timely access to farm machinery at affordable rates at the collective/group level. This particularly helped women farmers reduce drudgery (especially in weeding and harvesting in both groundnut and black gram), free up time for other tasks (in the absence of their migrant husbands) and importantly, provide a service which smallholders could not afford individually.

While for the nascent arrangements, recognition of their identity as farmers itself appeared as a growth need (given generations of deprivation and exploitation), in the case of the mature FPO, some of the initial struggles appeared to be forgotten, with issues of identity and autonomy presented as basic existence needs. Similarly, a transition was also observed from an internal focus, a ‘space of dependence’ to more external ‘spaces of engagement’. Yet there was also a cautionary note concerning this transition, and that was the need for continued engagement with the internal base, as this served as the foundation for any external engagement, both in material terms of capital contribution and social skills and network.

**Fig. 4:**
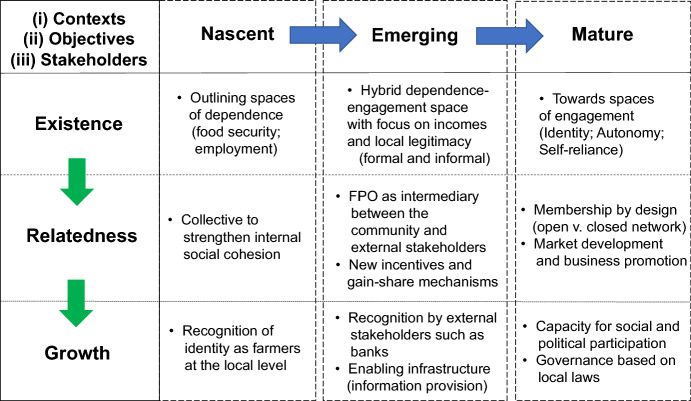
Principles to guide FPO design and assessment at various stages of development (Based on conceptual model)

Figure 4 summarises such transitions from individual needs to the group level across the three FPO stages. The supply network literature (Lee and Tang 2017) offers valuable research direction here; in its combined exploration of three dimensions: (1) *contexts* (emerging and developing economies); (2) *objectives* (economic, environmental, and social responsibility); and (3) *stakeholders* (producers, consumers, shareholders, social enterprises, institutions, and NGOs). Despite contextual differences, these guiding principles could be read as a continuum (in theory) as the needs of individuals were comparable within stages and often across stages too. Combining the pathways in horizontal (stages of development) and vertical (needs) directions, these exploratory insights can be used to inform more inclusive designs and assessment criteria, i.e. how support networks can be configured to deliver specific FPO social objectives (addressing RQ#2 in the study) featuring equity and inclusivity at its core.

Interestingly, in negotiating the collective spaces of SHGs, VOs and more formal FPOs or federations of groups, what emerged was a focus on people and their social relations vis-à-vis each other, and institutions of the state, as opposed to assessment metrics around compliance requirements. In practice, the informal *vyavastha* or MPAs appear well positioned to act as ‘spatial agents’ for establishing ethical norms (at the pre-registration stage) as FPOs collectivise and grow. This was observed in all three cases: in their nascent stages, informal MPAs emerged as ‘agents for change’, supported intensively by external agents, whether NGOs in Bihar and Tamil Nadu or a state programme in Uttarakhand. Their initial focus was internal, building group cohesion around shared interests, often through SHGs; providing space for evolving clusters of informal MPAs rather than just one.

For small producers, pathways to ‘maturity’ have proven to be lengthy with no guarantee that individual successes can be scaled or replicated (Poole and de Frece 2010). While some FPOs may be seen as ‘mature’, we argue that support networks never reach ‘maturity’. Emergence and transitions are constant—due to changing competitive priorities and emerging market opportunities. When working with poor, rural communities, with a low base of savings and capital, also differentiated by social identity (caste/ethnicity and gender), it is important to develop a flexible, bottom-up approach that can respond to needs as they arise. Given the lack of exposure of many rural women in Bihar (nascent case), we have observed, for instance, the importance of not taking collective action for granted, but rather working step-by-step with villagers to demonstrate the gains from such collectives. Even in the mature FPO, while recognising the benefits of membership, many women rued the lack of engagement at the same scale as in its initial years. We also now find some groups moving away to set up their own arrangements. Responsiveness to members’ local needs and interests, as they evolve, then needs to be a core ethical principle underlying FPO design, providing ‘*necessary context for the construction of spaces of engagement*’ (Cox 1998: 3).

A related issue is the co-existence of a diversity of systems, rather than a one-size-fits-all approach, as tends to often be the case in design. As this exploratory study has shown, there are often trade-offs between existence, relatedness and growth needs, with different arrangements prioritising different needs at various stages of their life cycle. While existence, or the need to see material benefits, often comes first for the poor, this is not the only goal; overcoming marginalisation and the stereotyping of their identities and capacities, also becomes important, more so, for rural women. These trade-offs need to be recognised and balanced, bringing the questions of needs versus administrative requirements to the fore.

## Conclusion

The farmer-producer organisation (FPO) is an umbrella term used to describe modes of farmer collectivisation in India. While women farmers play a central role in agriculture, they continue to be marginalised in terms of their participation in FPO activities and governance structures. Participation is no guarantee for empowerment if underlying social issues, that result in gender inequality, are not addressed (Mudege et al. 2017). This study, therefore, focused on social ‘intangibles’ as well as economically framing the idea of the FPO (i.e. benefitting from economies of scale and greater bargaining capacity), engaging with the collective action debate with a new and intersectional approach—based on specific members' needs and critical issues around social inclusion.

To address a specific gap in the literature around better framing societal needs and social inclusion, the dimensions of existence, relatedness and growth were conceptualised in terms of what they might mean for spaces of activity (engagement or dependence). We discussed the idea of FPOs as a collective-shaping continuum, where inclusivity and members’ needs are dynamic and dependent across stages of FPO development.

This research is novel—in presenting new empirical evidence of how women-led FPOs are establishing social norms and organising for equity and inclusivity, we contribute to the research agenda on gender-inclusive development. In informing a better understanding of the interplay between the needs of individuals and the idea of collective spaces offered by FPOs, our findings also contribute new insights into ways in which collective spaces are forming, the hierarchy of spaces and the kinds of nurture they require. While the advantages of FPOs in general are clear, distinctions across the many types and stages envisioned in the future are less so. This research reveals guiding principles on how to organise FPOs in different stages, in terms of social objectives. In promoting new forms of participation at various stages, these principles can provide valuable new criteria for designing more equitable and inclusive FPOs.

### Policy implications

Indian society is hugely differentiated by caste, ethnicity, class and gender, and strategies to address these differences to create more inclusive spaces need to be developed. This exploratory study can inform industrial and governmental policies and strategies to support nascent, emerging and mature FPOs in specific regional contexts. What is clear is the need for policy to be more attentive to issues of social equity, principles of inclusion and measures of fairness. These need to go beyond the annual turnover, contributions of members to the business, or dividend distribution, as set by state institutions that rate FPOs, to include an expansion in the ‘spaces of engagement’ of differently positioned people. In terms of achieving social objectives, this study’s new guiding principles are relational, involving collaborative and qualitative aspects, that might complement financial and quantitative metrics. While there is good evidence of FPOs meeting such social objectives, in the case of Tamil Nadu (emerging) and Uttarakhand (mature), both FPOs are struggling financially, as they look to meet the rating criteria, essential for accessing state resources.

One needs to think innovatively about the balance between working capital and decentralised management, saving time and reducing drudgery, particularly for women, expanding access to knowledge and information, that can ultimately generate a critical consciousness contributing to the development of equitable social institutions. These principles may help build multiple, interlinked strategies seeking ultimately to realign relationships of unequal power (with implications for future research around socially responsible supply networks).

### Limitations and future research

Women’s agency results from multiple factors that are dependent on context (e.g. socio-economic, cultural, political and environmental); hence, causal links are complex and vary as the context changes from case to case (Rao et al. 2019). A cross-case analysis/comparative perspective is problematic for various reasons, including differences in agrarian structure, technological advancement, geography and political economy factors. As this study is exploratory, it cannot control for these factors. However, it lays foundation for future work that could include using case-based comparative research techniques (e.g. qualitative comparative analysis) to address concerns of complex causality and high context dependence of data (Ragin 1987); or quantitative studies based on large-scale surveys to test and refine the conceptual framework across more FPO sites and contexts.

## Figures and Tables

**Figure:**
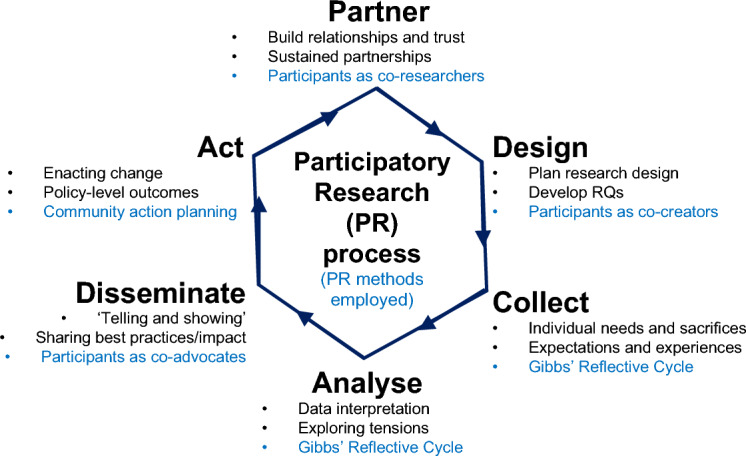
Participatory research process (adapted from Vaughn and Jacquez 2020)

## Appendix 1: Ethical considerations, participatory research (PR) process and Interview protocol

Ethical aspects involving research interview protocols carried out as part of the TIGR^2^ESS programme were approved by the International Development Ethics Committee at an academic institution in the UK. With the permission of participants, the anonymity of the data was achieved by adopting the use of pseudonyms as opposed to using real names. All participants were requested to give their consent to take part in interviews after researchers provided them with detailed information on the PR process and interview procedures and how data was going to be used. Participants were also informed that they could withdraw from the study at any time. Requests can be made to the lead author for access to selected raw data (some data relating to this publication cannot be openly released as the interviewees did not consent to open data sharing).

### Part 1. Context setting

- Discussion about user profile; background, specific objectives, and mission statements of FPO (i.e. Nascent–Emerging–Mature stories)
- Introduction to the PR Process, ERG theory and Gibbs Reflective Cycle (1988)
- Statement of confidentiality: “*All your responses will be anonymised and all information that could permit the identification of you will be regarded as strictly confidential. It will be used for this research only and will not be released or disclosed without your prior consent. You can withdraw your participation at any point in this study*”.

### Part 2. Expectation

- In terms of 1a and 1b, why did you come together/why did you join the group?
- In terms of ‘**Existence’**, what were your expectations/priorities before involvement?
  Please prioritise and elaborate on (for example):
  1. Livelihood and food
  2. Protection, shelter and a better house to protect you against the elements
  3. To meet health needs
  4. To meet your need for a network or organisation that might protect you from violence and abuse
  5. To meet your need for a safe network or organisation that protects you from being cheated, and to access markets or inputs or other resources that you want
  6. Other
- In terms of **‘Relatedness’**, what were your expectations/priorities before involvement?
  Please prioritise and elaborate on (for example):
  1. Opportunities to get together in a group, for interaction and support
  2. For better health of soil, natural resources, plants, or the environment
  3. To learn new things, to gain more information and knowledge
  4. To have a chance to enjoy and appreciate the world around you without worries
  5. To have a comfortable life for you and your family, one without hardship
  6. Other
- In terms of ‘**Growth’**, what were your expectations/priorities before involvement?
  Please prioritise and elaborate on (for example):
  1. For our community/village to be known as farmers/for quality of the produce/or for women to be identified as farmers
  2. For our members to receive fair treatment, in terms of price and opportunity, regardless of who they are
  3. For returns to each person that collectively benefits the entire community, rather than only some people getting benefits and others getting left behind
  4. To have a say in matters of the collective/group, and community; to have a standing in the village/community where people recognise and acknowledge you, your contribution and status
  5. Freedom from other opinions; where you have the freedom to do what you want and go where you want to go
  6. Other

### Part 3. Experience

- To what extent were your expectations addressed by arrangements you were/are part of?
- What specific arrangements contributed most to addressing your expectations?
- What could have been done differently/better to meet your priorities?

### Part 4. Actions

1. What are your (new?) expectations from this particular arrangement (as it exists now)?
2. What specific arrangements could contribute to addressing your (new?) expectations?
3. What should be done differently in the future to meet your (new?) priorities?

## Appendix 2: Summary of commentaries on selected discriminating features at different stages of emergence

**Table:**

	Nascent Bihar Vyavastha Micro-Producer Arrangement (MPA)	Emerging Nallavur Agricultural Farmer-Producer Company (FPC)	Mature The Nari Ekta Self-Reliant Co-Operative
Commentaries on equity and inclusivity	Women do not own land. They do not view themselves as farmers, nor does the family or local milieu consider them more than family farm labour; there is a focus on building self-confidence and self-esteem, to participate in, visualise and lead any MPA. A key challenge in building equity and inclusivity in nascent stages of mobilisation is self-exclusion. Any movement to participate or lead requires critical consciousness and confidence among the women. It also requires males to be open to and to acknowledge women as equal if not as leaders. Inclusivity and equity were promoted by initiating the MPA within the space created by the women’s SHG network The strengths of the SHG network were that poor women, from scheduled tribe and socially disadvantaged backgrounds, would be prepared to engage with MPAs. They are organised, have an appetite for change, and are acknowledged locally by men and women as successful change agents. They also have the experience of working with local government actors and are identified as being effective at liaising with government, and in advocating.	Equity issues are at both gender (men and women) and class levels (small and marginal farmers vs big farmers); in terms of gender, women have less access to productive resources—land, knowledge, technology, finance, and markets; small and marginal farmers have less area under cultivation with challenges to access finance, market and needs-based technological support Inclusivity was promoted on the dimensions of gender, class and caste; Equal opportunities are ensured for marginalised sections of the community by evolving specific policies aimed at groups/collectives to access services and enable equitable participation in all decision-making processes.	Access to information, techniques, finance, market, and productive resources including land is very much influenced by gender, economic class, caste and also remoteness (distance from road and federation centre) Efforts to adopt an equitable approach for the inclusion of the most marginalised in women's groups are focused on: involvement in value-addition processes, income generation activities, awareness and capacity building and programme decision-making processes; Plans are underway to develop strategies for more inclusivity through the framing of plans and policies and programmes at various levels.
Commentaries on social inequalities and how they are being tackled in collective spaces	Apart from gender and class (discussed above), the specific social inequality in this site is the one related to indigenous or tribal communities. Tribals/Santals here are marginalised as communities and alienated from the mainstream. They see themselves as being lesser, and having to emulate the mainstream populations. Mutual trust with Dikus, or mainstream Hindu populations, was a key issue; With the initiators and adopter farmers being distinguished, there was a pull for tackling these inequalities. The driver was to increase the membership base, regardless of ethnicity, and the initiators went out of their way to mobilise membership. As Santal and non-Santal males and females worked together, the collective space was shaped.	A specific example is gender inequality: although women are contributing more to agricultural production, recognition is very limited. Specifically, external recognition by formal financial institutions (banks) and government departments to access entitlement schemes in agriculture; As a collective, this has been enabled and banks have now provided credit cards to women farmers and the Dept of Agriculture has provided scheme benefits to women farmers when approached as a collective. As individuals, farmers didn’t have the capacity and time to invest in such activities, even if access to services had been possible (which it wasn’t). The benefits of the collective approach now mean women farmers are leasing land for cultivation and expanding their production.	A specific example is inclusion; small and marginal farmers did not have the knowledge or confidence to access services and entitlements, nor an ‘identity’ Members in women's groups have tried to prioritise the distribution of benefits (e.g. grants, resources, services) based on economic class, caste, and marital status, at the group and federation level, and looking to integrate into plans and policies; As a result, collective spaces have provided women with an identity and they are recognised, in the block, district and financial institutions by the name of their federation Many women have made decisions at their level (unlike earlier times) about educational institutions for their children, and crop cultivation, especially for selling to the unit/market. Decision-making even at the household level was not very visible but is taking place very gradually.
Commentaries on transitioning from individual to group level needs (mutual trust and sacrifices)	The arrangements or vyavastha were instigated by individuals, male and female, Santal and non-Santal—in their villages and neighbouring areas. They went beyond their individual needs, to encourage adoption. They advocated for adoption with individuals in the village, and also with the office bearers of the SHG Village Organisations. For example, initiators took potential adopters and VO office bearers on visits to their farms, openly sharing knowledge in terms of techniques and potential, and provided working capital to cultivate new (riskier) crops. This built confidence and mutual trust with the adopters and others.	Positive social behaviour, based on mutual trust and continuous engagement, was central to collective action; these positive relationships were built through interactions which reduced a sense of vulnerability among its members. For example, in evidencing collective strength to access credit from banks, to aggregate produce to reach primary markets, to invest in common value addition centres and by learning together when it came to new technologies In terms of the sacrifices made, here the leaders of collectives spend additional time in coordination and management, taking part in key decision-making and executing different services to members. Taking on leadership positions has been very time-consuming, however, in spending their time for the common cause, the important trade-off is social identity and respect earned as leaders at the village level.	Women feel it to be a continuous learning process as they report that they have learned a lot (since the beginning of the formation of groups) about best practices, difficulties and issues which women from different sections and villages had been experiencing (knowledge sharing); they continue to work together to aggregate their produce, including those who live remotely or have less produce; deriving selling and purchase rates, and ensuring safe transport of their produce to the unit and other locations In terms of mutual trust and sacrifices, they have collectively made decisions to choose the neediest and marginalised woman/household for grants and sometimes collect money to pay on behalf of a poor single mother or woman-headed household.; They take time to participate in groups and federation meetings despite being very busy as they realise the importance of being part of decision-making processes. They feel satisfied and happy with improved awareness. confidence and improved livelihood opportunities these effort has brought. There is a realisation that a lot more effort needs to go in for the benefits to reach every household in the federation (and they are prepared to act).
Commentaries on new forms of participation	New forms of participation here began with the impetus for mobilisation coming from the initiators in the community rather than from the external agency, the NGO or government body. This extension from local actors, the initiator, was a key differentiator of this arrangement/mobilisation. As the instigation was locally driven, the governance and oversight role of the SHG network was critical. The SHG leadership had an overview of the processes and ensured that conflicts of interest were carely managed. The risks of slipping into a ‘dependent space’ were thus mediated; The overcoming of social barriers—gender and ethnicity—and the creation of a space inclusive of initiators was also a new form of participation. The power dynamics of the local milieu did not translate to MPAs, and access to information and transparency were maintained within and between the actors involved.	Here women are now participating from the position of the ‘farmer’, engaged in managerial roles, and making independent decisions which is very different from their previous contribution to production (as labour); From a managerial role perspective, decision-making processes have also been wide-ranging across the value chain: from crop selection to marketing and handling income.	Women are participating in new roles involving: value-addition processes, pricing decisions, distribution of important nutrition supplements to the most vulnerable sections of the community, making decisions to cultivate nutritious crops which were on a decline and thereby arresting their extinction; they are also exploring produce /products which could be produced through value addition and improving their businesses; While they continue being the main producers/farmers, members in the federation are now contesting local elections; As a collective, they even came together to file legal cases in response to policy decisions which could drastically impact the attempts of various small federations looking to be self-sufficient.
Commentaries on best practices advocated	What can nascent stage FPOs learn from the emerging and mature stage FPO cases? From the emerging FPO case, the learning is that focus on technical and management skills and market linkages needs to be broad-based within the group. Transparency in roles and transactions needs to be built in ab initio so that there is a strong foundation once formalised, and management and governance functions need to become external facing and open to scrutiny; From the mature case, the lesson is that social exclusion and mobilisation of members inclusively and equitably need to be built into the systems and processes of functioning. It cannot be opportunistic just to get started. Social mobilisation and sensitivity to intersectional ties need to be a management and governance task. Commodity-focused interventions are truncated and inadequate, and the focus needs to be on structures, systems and processes that instigate and support the FPO to be agile and meet member needs.	The key learning from emerging FPOs is to focus on the scale of operations and the engagement of members in business contribution, as well as human resources to manage the collectives; In nascent stage FPOs—best practices advocated include a focus on skill empowerment and the group/collective space to support them to gain power-within, which helps to expand their access and institutional linkages.	The focus of the mature FPO has become very much business-oriented, especially with successful ventures. Moreover, with no financial support from any govt/other programmes, and with many products in the product line, working collectively on social issues has taken a backseat; For the nascent and emerging cases, there is a strong reminder to strengthen the focus on gender and social aspects in all activities alongside business operations, especially including most marginalised sections. Those living in highly remote areas are getting left out in the process due to their low access, hence, exploring ways to revive and strengthen regular contact and association is key to re-introduce values and principles of collective action).

## Footnotes

1 INR1 = 0.012 USD; INR 1 million = 12,040.66 USD.
